# High prevalence of gonadal dysfunction in pediatric sarcoma survivors: insights from hormonal and clinical markers

**DOI:** 10.3389/fped.2026.1914861

**Published:** 2026-09-07

**Authors:** Selma Cakmakci, Aylin Kilinc Ugurlu, Nadide Basak Gulleroglu, Derya Ozdemir Tas, Cigdem Bulut, Mehmet Caner Ozer, Arzu Yazal Erdem, Derya Ozyoruk, Fatih Gurbuz, Mehmet Boyraz, Inci Ergurhan Ilhan, Neriman Sari

**Affiliations:** 1 Department of Pediatric Hematology and Oncology, Ankara Bilkent City Hospital, Ankara, Türkiye; 2 Department of Pediatric Endocrinology, Gazi University Faculty of Medicine, Ankara, Türkiye; 3 Department of Radiology, Ankara Bilkent City Hospital, Ankara, Türkiye; 4 Center for Assisted Reproduction, Ankara City Hospital, Ankara, Türkiye; 5 Department of Pediatric Hematology and Oncology, Tekirdag Ismail Fehmi Cumalioglu City Hospital, Tekirdag, Türkiye; 6 Department of Histology and Embryology, Health Sciences University Gulhane Faculty of Medicine, Ankara, Türkiye; 7 Department of Pediatric Endocrinology, Ankara Bilkent City Hospital, Ankara, Türkiye

**Keywords:** anti-Müllerian hormone, childhood cancer survivors, follicle-stimulating hormone, gonadal dysfunction, sarcoma

## Abstract

**Introduction:**

A critical proportion of childhood bone and soft tissue sarcoma survivors experience some form of reproductive impairment. These findings underscore the increasing need for a comprehensive evaluation of gonadal function in this patient population. This study aimed to evaluate gonadal function in survivors of childhood bone and soft tissue sarcomas and to identify treatment-related risk factors.

**Methods:**

This retrospective cohort study included survivors aged 17 years or older at evaluation who had received curative-intent treatment for bone or soft tissue sarcomas diagnosed between January 2009 and February 2022. Demographics, clinical history, hormonal assays [follicle-stimulating hormone (FSH), luteinizing hormone, anti-Müllerian hormone (AMH), inhibin B, estradiol, testosterone], pelvic ultrasonography (females), and semen analysis (males) were recorded.

**Results:**

The cohort comprised 38 participants (median age 18.75 years); 17 males (44.74%) and 21 females (55.26%). Gonadal dysfunction was observed in 18 participants (47.4%), including low ovarian reserve or diminished AMH as the most common finding in females and azoospermia as the most frequent abnormality in males. Normal gonadal function was preserved in 20 participants (52.6%). FSH levels were significantly higher in the gonadal dysfunction group in both females (*p* = 0.004) and males (*p* = 0.007).

**Discussion:**

Childhood sarcoma survivors have a high prevalence of gonadal dysfunction, with notable impairments in ovarian reserve and spermatogenesis, marked by elevated FSH in both sexes. Routine surveillance, patient education on gonadotoxicity and infertility risks, and pre-treatment fertility preservation counseling are essential.

## Introduction

Over the past few decades, there have been dramatic improvements in survival in patients with childhood cancers, with approximately 80% of achieving long-term remission (1, 2). Bone and soft tissue sarcomas are particularly of interest in this population since they predominantly arise in the pediatric age and demonstrate 5-year survival rates ranging from 65% to 81%, depending on tumor type and location (3, 4). However, improved survival has been accompanied by a growing frequency of long-term sequelae, including gonadal dysfunction, which can profoundly impact fertility, development, and overall endocrine health in survivors (5, 6).

Gonadal impairment arises from the aggressive multimodal therapies required for sarcoma control, including surgery, chemotherapy, and radiotherapy. Chemotherapeutic agents, particularly alkylating agents such as cyclophosphamide and ifosfamide are well-documented to have gonadotoxic effects (7–9). These agents disrupt gametogenesis and somatic cell function in the gonads, leading to impaired spermatogenesis in males and diminished ovarian reserve in females (10, 11). High cumulative doses of cyclophosphamide equivalents have been associated with azoospermia and premature ovarian insufficiency (12, 13). Radiotherapy administered to the pelvic region (or total body) increases damage by direct effects on ovarian follicles or testicular tissue, and can result in irreversible sterility (14, 15). Age at exposure and genetic predisposition further exacerbate this vulnerability (16, 17).

Despite the rising number of childhood cancer survivors, data on the prevalence of gonadal dysfunction remain limited. It is estimated that 20%-44% of survivors experience some form of reproductive impairment, with males often more susceptible to permanent infertility due to alkylator exposure, while females may face early menopause or pregnancy complications (18, 19). There is a growing need for comprehensive assessments of gonadal function, including investigation of associated factors such as treatment modalities (20, 21).

This study aims to evaluate gonadal function in survivors of childhood bone and soft tissue sarcomas treated at our institution, examining hormonal profiles, clinical markers, and treatment-related risk factors, while investigating factors associated with gonadal dysfunction. We sought to provide additional data that could improve reproductive outcomes and the decision-making process regarding the therapeutic management of this vulnerable population.

## Methods

### Study design and setting

This study was designed as a retrospective cohort analysis and conducted at the Pediatric Hematology and Oncology Clinic of Ankara Bilkent City Hospital, Ankara, Turkey, with data based on comprehensive patient records spanning from January 2009 to February 2022. Throughout this period, patients had been diagnosed and treated for definitive sarcoma diagnoses and had been followed by subsequent assessments of gonadal health after treatment.

The study population consisted of pediatric patients who had been diagnosed with bone or soft tissue sarcomas who had successfully completed their oncologic treatment regimens. Eligible participants included individuals aged 17 years or older at the time of gonadal evaluation, as this age threshold was chosen to allow a more reliable assessment of post-pubertal gonadal endocrine function and reproductive outcomes. Diagnoses were histologically confirmed during the childhood or adolescence. All subjects had completed curative-intent treatment (chemotherapy with or without radiotherapy or surgery) at least 12 months prior to evaluation. Exclusion criteria included patients with pre-existing endocrine disorders (e.g., congenital hypogonadism or genetic syndromes affecting gonadal development), those with incomplete medical records or missing key gonadal assessment data, individuals with secondary malignancies or recurrent disease requiring additional gonadotoxic therapies, and patients with significant comorbidities (e.g., chronic kidney disease or neurological impairments). Data were collected retrospectively from electronic medical records, laboratory databases, and imaging archives at Ankara Bilkent City Hospital, supplemented by structured clinical interviews during follow-up visits. Data included demographic, clinical, hormonal, imaging, and reproductive parameters.

### Ethics

The study protocol was approved by the Clinical Research Ethics Committee of Ankara Bilkent City Hospital (Approval date: 23/07/2025, decision no: E2-25-1985). Owing to the retrospective design of the study, the requirement for informed consent was waived by the Ethics Committee. All procedures were conducted in accordance with the principles of the Declaration of Helsinki and its subsequent amendments. Participant identities were anonymized throughout the research process, and data were stored securely and accessed only by authorized personnel to protect privacy and confidentiality.

### Study groups and comparative parameters

Participants were categorized into two groups based on predefined gonadal evaluation criteria: normal gonadal function and gonadal dysfunction. Gonadal dysfunction was defined as the presence of abnormalities detected in at least one domain of gonadal assessment, including hormonal evaluation, reproductive function, gonadal imaging, or semen analysis, suggestive of impaired gonadal endocrine function and/or reduced fertility potential.

Comparative analyses were performed between the normal gonadal function group and the gonadal dysfunction group. Demographic and anthropometric parameters including age at evaluation, sex distribution, height, weight, and body mass index (BMI) were compared between groups (22). Disease-related characteristics such as sarcoma subtype (categorized as Ewing's sarcoma, osteosarcoma, rhabdomyosarcoma, or synovial sarcoma), age at diagnosis, follow-up duration, and presence of metastasis at diagnosis were also evaluated.

Treatment-related variables including chemotherapy protocol, cumulative cyclophosphamide equivalent dose (CED, calculated using established formulas to quantify alkylating agent exposure), pelvic or testicular radiotherapy exposure, radiotherapy dose (including dose in Gray) were compared between groups.

Pubertal and reproductive characteristics were also assessed. In females, these included age at thelarche, pubarche, menarche, menstrual cycle regularity, menstrual cycle length, menstrual bleeding duration and amount estimated by pad usage, pregnancy history, presence of children and breast Tanner staging (23). In males, reproductive assessment included pubertal staging, testicular volume measurement via orchidometer, penile length, and semen analysis findings when available. Family history of infertility was additionally recorded.

Hormonal evaluation included serum follicle-stimulating hormone (FSH), luteinizing hormone (LH), estradiol (E2), anti-Müllerian hormone (AMH), total testosterone, and inhibin B levels. Elevated gonadotropins, reduced sex steroid levels, diminished ovarian reserve markers, or impaired Sertoli/germ cell markers were considered suggestive of gonadal dysfunction.

Sex-specific subgroup analyses were also performed to further evaluate hormonal and reproductive outcomes separately in male and female participants.

### Hormonal assays

Gonadal function was evaluated through serum hormone measurements using standardized immunoassays. All blood samples were drawn in the morning. In menstruating females, blood draws were scheduled during the early follicular phase. All assays were performed in a certified laboratory with quality controls.

### Imaging studies

Pelvic ultrasonography was conducted by pediatric radiologists using high-resolution transabdominal probes to assess female reproductive anatomy. Measurements included uterine dimensions (longitudinal, anteroposterior, and transverse in mm), endometrial double-layer thickness (mm), and ovarian volumes (right and left ovaries in cm^3^) (24). These parameters were evaluated against age-matched norms.

### Semen analysis

In male participants, semen analysis was performed according to World Health Organization guidelines at the hospital's Assisted Reproductive Techniques Center. Parameters included sperm concentration, motility, and morphology to diagnose conditions such as azoospermia, oligoasthenoteratozoospermia, or seminiferous tubule injury. Samples were collected via masturbation after 2-5 days of abstinence and analyzed within one hour.

### Definition of gonadal dysfunction

Severe testicular dysfunction was defined by serum inhibin B levels < 80 pg/mL, particularly when associated with elevated FSH concentrations, reduced testicular volume, and azoospermia or severely impaired semen parameters (25–28). Elevated serum FSH was defined as >10 IU/L, consistent with previous male infertility studies (25, 26, 29). Ovarian dysfunction was considered in women with elevated serum FSH levels (>10 IU/L), reduced AMH concentrations (<1 ng/mL), and/or decreased ovarian volume (<4 mL) on pelvic ultrasonography (30–32).

### Statistical analysis

All analyses were performed on IBM SPSS Statistics for Windows, Version 27 (IBM Corp., Armonk, NY, USA). *p* < 0.05 values were accepted as statistically significant results. For the normality check, the Shapiro–Wilk test was used. Descriptive statistics were presented using mean ± standard deviation for normally distributed continuous variables, median (25th percentile—75th percentile) for non-normally distributed continuous variables and frequency (percentage) for categorical variables. Normally distributed continuous variables were analyzed using the Student's t test. Non-normally distributed continuous variables were analyzed using the Mann Whitney *U* test. Categorical variables were analyzed using the Fisher's exact test or Fisher-Freeman-Halton test.

## Results

### Study population and cohort characteristics

**Figure 1:**
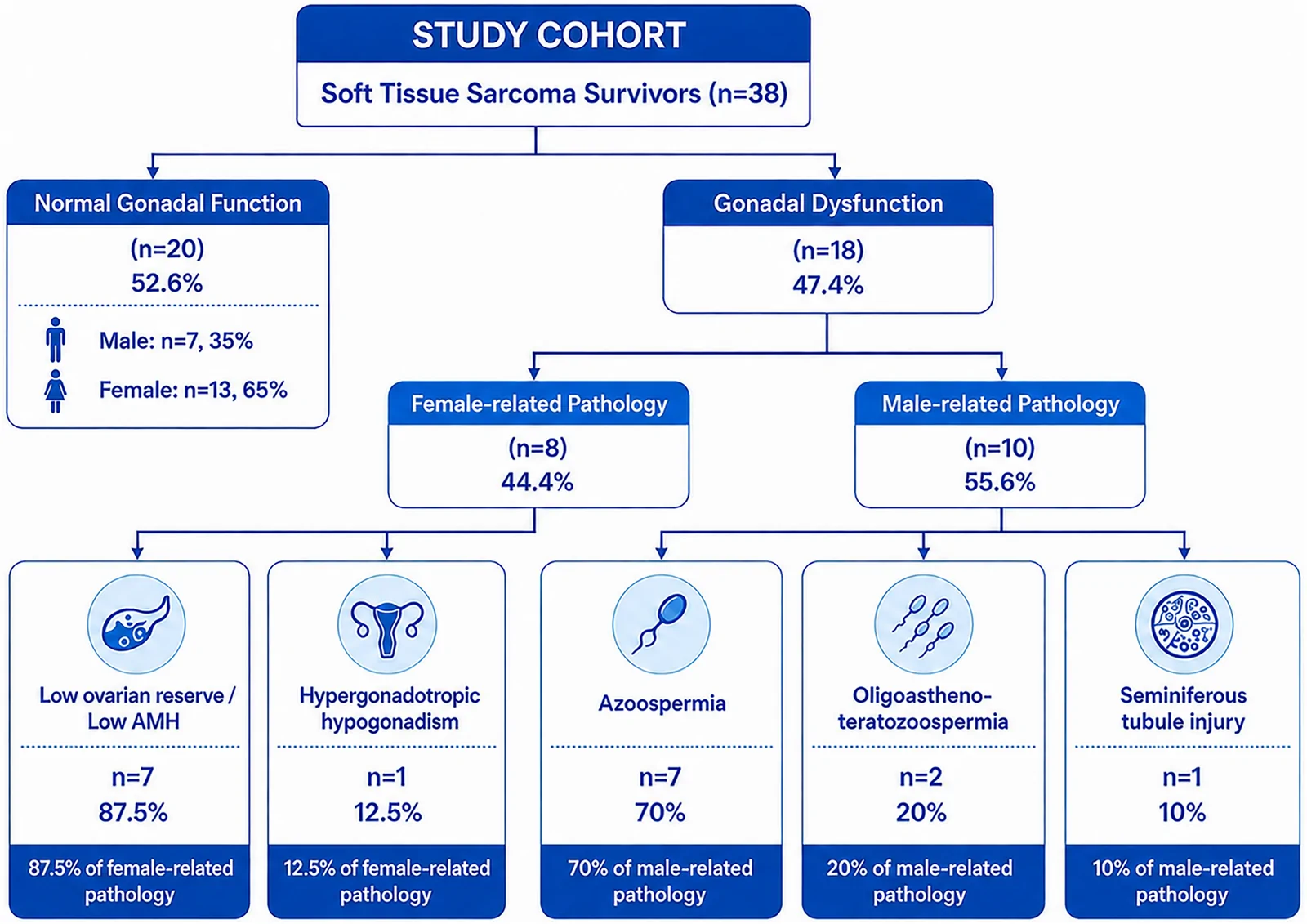
Distribution of gonadal function and reproductive pathologies in the study cohort (*n* = 38). AMH, anti-Müllerian hormone.

A total of 38 survivors of bone and soft tissue sarcoma underwent gonadal function evaluation after completion of cancer treatment. Among the entire cohort, normal gonadal function was observed in 20 patients (52.6%), whereas 18 patients (47.4%) demonstrated evidence of gonadal dysfunction (Figure 1). Within the gonadal dysfunction group, ovarian dysfunction was identified in 8 patients (44.4%), while testicular dysfunction was observed in 10 patients (55.6%). Among ovarian dysfunctions, low ovarian reserve/diminished AMH represented the predominant abnormality and was detected in 7 patients, accounting for 87.5% of ovarian dysfunction cases. Hypogonadotropic hypogonadism (central gonadal insufficiency) was detected in only 1 patient (12.5%). Among testicular dysfunctions*,* azoospermia was the most frequent abnormality, observed in 7 patients (70%), followed by low inhibin B/elevated FSH in 2 patients (20%) and reduced testicular volume in 1 patient (10%) (Figure 1).

### Demographic and anthropometric characteristics

The cohort consisted of 17 males (44.7%) and 21 females (55.3%). In the normal gonadal function group, 7 patients were male (35.0%) and 13 were female (65.0%), while in the gonadal dysfunction group, 10 patients were male (55.6%) and 8 were female (44.4%). No statistically significant difference was observed between the groups with respect to sex distribution (*p* = 0.203). Anthropometric measurements including height (*p* = 0.034), weight (*p* = 0.632), and body mass index (BMI) (*p* = 0.322) were evaluated between groups. Height was significantly greater in the gonadal dysfunction group compared to the normal gonadal function group (170.5 cm vs. 160.0 cm; U = 89.000, *p* = 0.034), while weight and BMI were comparable between groups (Table 1).

**Table 1:** Demographic and anthropometric characteristics according to gonadal dysfunction Status.

Characteristic	Total (*n* = 38)	Normal gonadal function (*n* = 20)	Gonadal dysfunction (*n* = 18)	*p*
Age, years	18.75 (17–21)	18.00 (17.9–22)	19.25 (17–20.7)	0.230^‡^
Sex				0.328^§^
Male	17 (44.74%)	7 (35.0%)	10 (55.6%)	
Female	21 (55.26%)	13 (65.0%)	8 (44.4%)	
Height, cm	168 (159–172)	160.0 (155–175)	170.5 (159.4–172)	**0** **.** **034** ^‡^
Weight, kg	64.60 ± 17.95	63.0 ± 21.45	59.4 ± 14.53	0.632^†^
Body mass index, kg/m^2^	22.49 (19.27–27.43)	22.67 (18.55–31.02)	19.80 (19.28–26.87)	0.322^‡^

^†^Student's *t* test.

^‡^Mann–Whitney *U* test.

^§^Fisher's exact test.

### Disease-related characteristics

The overall distribution of sarcoma subtypes did not significantly differ between groups (*p* = 0.332). The median age at cancer diagnosis was significantly higher in the gonadal dysfunction group compared to the normal gonadal function group [15 years [IQR: 11–15.3] vs. 11 years [IQR: 10.1–14.3]; *p* = 0.015]. The duration of follow-up was similar between groups [95.0 months [IQR: 26.0–143.3] vs. 49.6 months [IQR: 21.8–178.2]; *p* = 0.082]. Metastatic disease at diagnosis was observed in 6 patients (15.8%) and occurred at comparable rates in both groups (10.0% vs. 22.2%; *p* = 0.395) (Table 2).

**Table 2:** Disease-Related and treatment characteristics according to gonadal dysfunction Status.

Characteristic	Total (*n* = 38)	Normal gonadal function (*n* = 20)	Gonadal dysfunction (*n* = 18)	*P*
Diagnosis (*n* = 38)				0.332^¶^
Ewing's sarcoma	13 (34.21%)	5 (25.0%)	8 (44.4%)	
Osteosarcoma	17 (44.74%)	10 (50.0%)	7 (38.9%)	
Rhabdomyosarcoma	7 (18.42%)	5 (25.0%)	2 (11.1%)	
Synovial sarcoma	1 (2.63%)	0 (0.00%)	1 (5.6%)	
Age at diagnosis, years	14 (10.2–15)	11 (10.1–14.3)	15 (11–15.3)	**0** **.** **015** ^‡^
Follow-up time, months	75.15 (36.60–114.99)	95.0 (26.0–143.3)	49.6 (21.8–178.2)	0.082^‡^
Metastasis at diagnosis	6 (15.79%)	2 (10.0%)	4 (22.2%)	0.395^†^
CED, g/m^2^, median (range)^a^	22.14 (3.4–30.8)	22.14 (3.4–30.8)	23.17 (13.1–28.3)	0.977^‡^
Pelvic/Testicular RT				0.595^†^
No	35 (92.1%)	19 (95.0%)	16 (88.9%)	
Yes	3 (7.89%)	1 (5.0%)	2 (11.1%)	

Patients treated exclusively with cisplatin (*n* = 14) were not included in the CED analysis.

a CED analysis included 24 patients exposed to alkylating agents (12 with normal gonadal function and 12 with gonadal dysfunction).

† Fisher's exact test.

‡ Mann–Whitney *U* test.

¶ Fisher–Freeman–Halton test.

### Treatment-related characteristics

Chemotherapy regimens varied among patients, with the methotrexate, doxorubicin, and cisplatin (MAP) protocol being the most frequently used regimen, followed by EURO-EWING 99 (EE99) and vincristine, actinomycin-D, and cyclophosphamide (VAC)-based protocols. No significant difference in chemotherapy protocol distribution was observed between the gonadal dysfunction and normal gonadal function groups (*p* = 0.706).

The cumulative cyclophosphamide equivalent dose (CED) did not significantly differ according to gonadal function status (*p* = 0.977). Pelvic or testicular radiotherapy had been administered in a limited number of patients (7.9%, *n* = 3), and radiotherapy exposure rates were similar between groups (5.0% vs. 11.1%; *p* = 0.595). Due to the very small number of patients who received radiotherapy (*n* = 2 with available dose data), a formal comparison of radiotherapy doses between groups was not performed (Table 2).

### Pubertal and reproductive characteristics

Among female participants (*n* = 21), the median age at breast development was 9.5 years (range: 8.0–13.0), with no significant difference between groups (*p* = 0.117). The median age at pubic hair development was 11 years (range: 10–13) in the normal gonadal function group and 12.5 years (range: 11–13) in the gonadal dysfunction group, with a statistically significant difference between groups (*p* = 0.032). The median age at menarche was 12.0 years (IQR: 11.0–13.0) in the normal gonadal function group and 14.0 years (IQR: 13.0–15.0) in the gonadal dysfunction group; however, this difference did not reach statistical significance (*p* = 0.090).

Irregular menstrual cycles were reported in 5 patients (26.3%) and were significantly more frequent in the gonadal dysfunction group compared to the normal gonadal function group (57.1% vs. 8.3%; *p* = 0.038). Menstrual cycle length did not significantly differ between groups (*p* = 0.510). Menstrual bleeding duration (*p* = 0.699) and bleeding amount estimated by pad usage (*p* = 0.917) were also comparable between groups.

Only one female participant in the normal gonadal function group reported a history of pregnancy (*p* = 1.000), and no participants had children. A family history of infertility was reported in one patient in the normal gonadal function group, with no significant intergroup difference (*p* = 1.000).

Pubic hair Tanner staging was predominantly stage 5 in both groups (83.3% vs. 87.5%), with no significant difference (*p* = 1.000). Similarly, breast Tanner stage 5 was observed in the majority of female participants in both groups (91.7% vs. 100.0%; *p* = 1.000).

Among male participants (*n* = 17), testicular volume was lower in the gonadal dysfunction group compared to the normal gonadal function group [13.5 mL [8–20] vs. 18.5 mL [12–25]], although this difference did not reach statistical significance (*p* = 0.499). Penile length was also comparable between groups (*p* = 0.100) (Table 3).

**Table 3:** Pubertal and reproductive characteristics according to gonadal dysfunction Status.

	Total (*n* = 38)	Normal gonadal function (*n* = 20)	Gonadal dysfunction (*n* = 18)	*p*
Female participants (*n* = 21)				
Age at pubic hair development, years	11 (10–13)	11 (11–11)	11 (10–13)	0.825^‡^
Age at breast development, years	9.5 (8.0–13.0)	9.0 (8.0–13.0)	12.0 (11.0–13.0)	0.117^‡^
Age at menarche, years	12.5 (11.0–15.0)	12.0 (11.0–13.0)	14.0 (13.0–15.0)	0.090^‡^
Irregular menstrual cycle	5 (26.3%)	1 (8.3%)	4 (57.1%)	0.038^†^
Menstrual cycle length, days	28.5 (25.0–60.0)	28.0 (25.0–30.0)	45.0 (30.0–60.0)	0.510^‡^
Menstrual bleeding, days	6.5 (3.0–8.0)	6.0 (5.0–7.0)	5.0 (5.0–7.0)	0.699^‡^
Menstrual bleeding amount, pads	2.0 (1.0–4.0)	2.0 (2.0–3.0)	2.5 (1.0–4.0)	0.917^‡^
Pregnancy history	1 (4.8%)	1 (7.7%)	0 (0.0%)	1.000^†^
Infertility history in family	1 (4.8%)	1 (7.7%)	0 (0.0%)	1.000^†^
Tanner stage, pubic hair				1.000^†^
Stage 4	3 (15.0%)	2 (16.7%)	1 (12.5%)	
Stage 5	17 (85.0%)	10 (83.3%)	7 (87.5%)	
Tanner stage, breast				1.000^†^
Stage 4	1 (5.0%)	1 (8.3%)	0 (0.0%)	
Stage 5	19 (95.0%)	11 (91.7%)	8 (100.0%)	
Male participants (*n* = 17)	Normal Gonadal Function (*n* = 7)		Gonadal Dysfunction (*n* = 10)	
Testicular volume, mL	13.5 (8–25)	18.5 (12.0–25.0)	13.5 (8.0–20.0)	0.499^‡^
Penis length, cm	12.5 (4–15)	14.0 (13.0–15.0)	11.0 (4.0–13.0)	0.100^‡^

^†^Fisher's exact test.

^‡^Mann–Whitney *U* test.

### Hormonal findings

Serum follicle-stimulating hormone (FSH) levels were significantly higher in patients with gonadal dysfunction compared with those with normal gonadal function in both sexes. Among females, FSH was significantly elevated in the gonadal dysfunction group (*p* = 0.004), and among males, this difference was also statistically significant (*p* = 0.007).

Luteinizing hormone (LH) levels did not significantly differ between groups in females (*p* = 0.082) or males (*p* = 0.425). Estradiol (E2) levels were also comparable among female participants (*p* = 0.070). Anti-Müllerian hormone (AMH) levels were significantly lower in females with gonadal dysfunction [0.31 ng/mL [0.03–2.82] vs. 1.01 ng/mL [0.23–4.30]; *p* = 0.025].

Total testosterone levels did not significantly differ between groups (*p* = 0.394). Likewise, inhibin B concentrations showed no statistically significant difference according to gonadal dysfunction status (*p* = 0.217) (Tables 4, 5).

**Table 4:** Hormonal and ultrasonographic parameters in female participants according to gonadal dysfunction Status.

Parameter	Total (*n* = 21)	Normal gonadal function (*n* = 13)	Gonadal dysfunction (*n* = 8)	*p*
Hormonal Parameters				
FSH, mIU/mL	5.90 (2.20–61.7)	4.50 (2.20–9.40)	12.50 (2.50–61.7)	**0** **.** **004** ^‡^
LH, mIU/mL	3.90 (0.90–27.0)	3.20 (1.50–26.0)	9.15 (0.90–27.0)	0.082^‡^
E2, pg/mL	73.0 (28.0–198.0)	73.0 (37.0–293.0)	55.0 (11.8–187.0)	0.070^‡^
AMH, ng/mL	0.66 (0.03–4.30)	1.01 (0.23–4.30)	0.31 (0.03–2.82)	**0** **.** **025** ^‡^
Uterine and Ovarian Measurements				
Uterine longitudinal diameter, mm	67.0 (36.0–92.0)	70.0 (50.0–92.0)	55.0 (36.0–72.0)	0.174^‡^
Uterine endometrial thickness, mm	6.0 (1.0–13.0)	6.25 (2.0–13.0)	4.50 (1.0–10.0)	0.612^‡^
Right ovarian volume, cm^3^	5.90 (1.80–30.0)	7.85 (3.18–30.0)	2.80 (1.80–8.0)	0.050^‡^
Left ovarian volume, cm^3^	6.50 (2.50–12.8)	7.25 (2.90–12.8)	3.10 (2.50–7.0)	**0** **.** **034** ^‡^

^‡^Mann–Whitney U test.

**Table 5:** Hormonal parameters in male participants according to gonadal dysfunction Status.

Parameter	Total (*n* = 17)	Normal gonadal function (*n* = 7)	Gonadal dysfunction (*n* = 10)	*P*
FSH, mIU/mL	7.50 (1.20–14.0)	4.30 (1.20–6.60)	8.00 (7.20–14.0)	0.007^a^
LH, mIU/mL	5.80 (2.80–7.20)	4.40 (3.40–6.10)	5.90 (2.80–7.20)	0.425^a^
Total testosterone, ng/mL	4.08 (2.19–8.64)	3.36 (2.75–5.80)	4.80 (2.19–8.64)	0.394^a^
Inhibin B, pg/mL	78.5 (15.0–260.0)	93.5 (63.5–260.0)	38.0 (15.0–213.0)	0.217^a^

FSH was available in 15 males (*n* = 5 normal, *n* = 10 dysfunction); testosterone in 10 males (*n* = 4 normal, *n* = 6 dysfunction); inhibin B in 13 males (*n* = 4 normal, *n* = 9 dysfunction).

Descriptive statistics are presented using mean ± standard deviation for normally distributed continuous variables, median (25th percentile—75th percentile) for non-normally distributed continuous variables and frequency (percentage) for categorical variables.

AMH, anti-müllerian hormone; E2, Estradiol; EE2012, EURO-EWING 2012 protocol; EE99, EURO-EWING 99 protocol; FSH, Follicle-stimulating hormone; Gy, Gray; HRRMS, High-Risk Rhabdomyosarcoma protocol; IVADO, Ifosfamide vincristine actinomycin D doxorubicin; kg, kilogram; LH, Luteinizing hormone; MAP, Methotrexate doxorubicin cisplatin; MAP-IFO, Methotrexate doxorubicin cisplatin ifosfamide; NCI, National Cancer Institute protocol; RT, Radiotherapy; VAC, Vincristine actinomycin D cyclophosphamide; HDIFO, High dose ifosfamide.

a Mann Whitney *U* test. Data presented as median (min–max).

### Imaging findings

Pelvic ultrasonographic evaluation revealed that uterine longitudinal diameter was comparable between groups (*p* = 0.174). Uterine endometrial thickness did not significantly differ between patients with and without gonadal dysfunction (*p* = 0.612). Right ovarian volume tended to be lower in the gonadal dysfunction group, approaching statistical significance [2.80 cm^3^ [1.80–8.0] vs. 7.85 cm^3^ [3.18–30.0]; *p* = 0.050], while left ovarian volume was significantly reduced in the gonadal dysfunction group compared to the normal gonadal function group [3.10 cm^3^ [2.50–7.0] vs. 7.25 cm^3^ [2.90–12.8]; *p* = 0.034] (Table 4).

### Semen analysis findings

Semen analysis was performed in 12 of 17 male participants; 5 patients were unable to provide a semen sample. Among those analyzed, azoospermia was the most common finding, identified in 7 patients (58.3%), followed by normal semen parameters in 3 patients (25.0%). Oligoasthenozoospermia was detected in 2 patients (16.7%). One patient who was unable to provide a semen sample had a history of cryptorchidism operated at the age of 4 years and was classified as having seminiferous tubule injury based on hormonal evaluation.

## Discussion

The current study revealed that nearly half of childhood sarcoma survivors exhibited gonadal dysfunction, with the remaining demonstrating preserved gonadal function. Low ovarian reserve or diminished AMH levels were the most common findings in females, and azoospermia or oligoasthenoteratozoospermia were common among males. Rare but notable findings also included hypogonadotropic hypogonadism among females and seminiferous tubule injury among males. FSH levels appeared to be associated with gonadal dysfunctions, particularly in both sexes. Among demographic variables, the age at cancer diagnosis was significantly higher in patients with gonadal dysfunction, suggesting that pubertal or peripubertal exposure to gonadotoxic agents may confer greater susceptibility to gonadal damage, consistent with observations that older age at treatment is associated with more pronounced gonadal impairment (33–35). Patients with gonadal dysfunction were significantly taller, which may reflect delayed epiphyseal closure secondary to sex steroid deficiency (3). An additional consideration is the significantly older age at cancer diagnosis observed in the gonadal dysfunction group. Older age at diagnosis may reflect differences in baseline pubertal status and growth dynamics before initiation of oncologic therapy, potentially influencing attained adult height independent of treatment-related gonadal dysfunction. Therefore, the observed height difference is likely multifactorial and may result from the combined effects of baseline growth characteristics, age at diagnosis, and subsequent impairment of gonadal function. This should be considered when interpreting the association between gonadal dysfunction and final height. No other clinical variables, such as treatment duration and follow-up time, were found to differ significantly between groups.

Pelvic ultrasonography demonstrated a significantly lower left ovarian volume in patients with gonadal dysfunction. Reduced ovarian volume was generally accompanied by low AMH concentrations, suggesting that this imaging finding may reflect diminished ovarian reserve. In particular, two patients with markedly reduced bilateral ovarian volumes had hypergonadotropic hypogonadism and very low AMH levels, a pattern consistent with primary ovarian injury and possible depletion of the primordial follicle pool. However, another patient with reduced ovarian volumes had hypogonadotropic hypogonadism, indicating that inadequate gonadotropin stimulation may also contribute to reduced ovarian size. Therefore, ovarian volume should not be interpreted as a specific marker of chemotherapy-induced follicular depletion in isolation. Moreover, because antral follicle count was not assessed, the relationship between ovarian volume and the remaining follicular pool could not be directly evaluated. The small sample size and the finding of statistical significance only for the left ovary also warrant cautious interpretation.

Gonadal dysfunction remains a prevalent long-term sequela in survivors of childhood cancers, but reported frequencies vary widely due to differences in treatment protocols and assessment methods (1, 2). For instance, alkylating agents and radiation therapy are well-established risk factors for reproductive impairment, as they disrupt gametogenesis and somatic gonadal cell function, which are crucial properties that impact not only fertility but overall hormonal development (3, 4). Our findings indicate that approximately 47% of survivors displayed gonadal abnormalities, which aligns with observations in broader childhood cancer survivor cohorts where gonadal toxicity is heightened in relation to hematopoietic stem cell transplantation or platinum-based therapies (5). For example, in a recent systematic review by Weidlinger et al. (36), suspected infertility was noted in 5.1% of female osteosarcoma survivors and 13.0% of female Ewing sarcoma survivors, with higher risks linked to alkylating agents; this contrasts with our higher overall rate, potentially due to the inclusion of both sexes and a focus on sarcomas involving intensive regimens. Similarly, in a prospective study by Mörse et al. (33), young females treated for Ewing sarcoma exhibited profound declines in AMH shortly after treatment initiation, with no recovery in most cases except the youngest patients, mirroring our observations of persistent ovarian reserve impairment and emphasizing the early onset of gonadotoxicity in this subgroup. These contemporary findings underscore the need for early gonadal function screening in survivors to mitigate fertility risks, particularly in sarcoma cases where multimodal therapies amplify gonadotoxic effects through cumulative alkylator exposure and potential radiation scatter.

Sex-specific differences in gonadal vulnerability are evident in childhood cancer survivors, with males often experiencing more severe and persistent reproductive impairment compared to females (10, 11). This disparity may stem from the continuous nature of spermatogenesis vs. the finite ovarian follicle pool, rendering males more susceptible to cytotoxic insults (12). In our study, FSH elevations were particularly pronounced in both sexes with gonadal dysfunction, consistent with disrupted Sertoli cell function and seminiferous tubule integrity in males, and with diminished ovarian reserve in females. Comparable results were reported in a mega-analysis by Yano Maher et al. (34), where AMH levels in female childhood cancer survivors correlated with treatment risk and pubertal status, with peripubertal or older patients showing lower recovery; this suggests that pubertal timing influences resilience, as prepubertal gonads may benefit from relative quiescence, consistent with our finding that older age at cancer diagnosis was associated with greater gonadal impairment. Serum AMH concentrations continue to increase throughout late adolescence before reaching peak values during early adulthood. Therefore, AMH should be interpreted cautiously in adolescent cancer survivors, particularly in those who have not yet reached full reproductive maturity. In our cohort, reduced AMH levels were evaluated together with gonadotropins, ovarian ultrasonography, and clinical findings rather than being considered an isolated indicator of ovarian reserve. In our sarcoma-specific group, we found that males might be disproportionately affected (17). Physiologically, male germ cells undergo rapid proliferation, making them prime targets for chemotherapy-induced apoptosis, whereas female oocytes may exhibit partial resistance but accumulate DNA damage leading to accelerated atresia (18, 19). A systematic review by Steinmann et al. (35) further supports this, reporting suspected infertility in 71.4% of female and 60.3% of male soft tissue sarcoma survivors, emphasizing the role of chemotherapy in both sexes and highlighting that alkylating agents like cyclophosphamide contribute to dose-dependent Sertoli cell damage in males and primordial follicle loss in females. Recent perspectives on immune dynamics and sex hormone interactions throughout life further contextualize these sex differences, suggesting that hormonal milieu may modulate gonadal recovery patterns in a gender-specific manner (37).

Treatment modalities, particularly the intensity of chemotherapy and radiation, are crucial in determining gonadal outcomes in childhood sarcoma survivors, with evidence suggesting dose-dependent risks (20, 38). Our analysis showed no significant intergroup differences in variables like cyclophosphamide equivalent dose or pelvic radiation. This is similar to a retrospective observational study by Urakawa et al. (3), where autologous hematopoietic stem cell transplantation and platinum agents were linked to gonadal dysfunction, with recovery more likely in those with lower cumulative exposures. In another cross-sectional evaluation by Jin et al. (4), female adolescent and young adult survivors treated with alkylators showed diminished ovarian reserve in 27.5%, with pelvic irradiation and stem cell transplantation as key predictors; our lack of association may reflect uniform high-risk protocols in sarcomas, masking dose effects. We found preserved function in 52.6% of survivors despite exposure, potentially due to lower doses or younger age at treatment, as prepubertal gonads may exhibit relative quiescence (21). Alkylators like ifosfamide can alkylate DNA in proliferating cells, inducing cross-links that trigger cell cycle arrest and apoptosis in germ and somatic lines (39, 40). Radiation ionizes cellular components, generating free radicals that damage DNA, with ovarian follicles particularly sensitive due to their limited regenerative capacity (41). In males, Sertoli cell dysfunction impairs spermatogonial support, leading to elevated FSH as a compensatory response (42). However, we observed no significant differences between survivors with and without gonadal dysfunction in terms of specific chemotherapy regimens or radiotherapy exposure, which may be explained by numerous factors including limited sample size. Recent data from Hou et al. (43) in hematopoietic stem cell transplant survivors reinforce this, showing high premature ovarian failure rates (88.2%) associated with cyclophosphamide equivalent doses, highlighting the cumulative impact of alkylators in hematological and sarcoma contexts, where conditioning regimens often exceed thresholds for irreversible damage. Optimal management of primary ovarian insufficiency in cancer survivors, including multidisciplinary approaches to hormone replacement, fertility counseling, and long-term monitoring, is therefore essential to address these risks effectively (44).

The study has several notable limitations that warrant cautious interpretation of the findings. The small sample size of 38 participants and single-center design restrict generalizability. The retrospective design introduces potential biases, which could underestimate the true prevalence of gonadal dysfunction. The observed height difference between groups may reflect the confounding effect of age at diagnosis rather than gonadal dysfunction *per se*. The evaluation at a median follow-up of approximately 7 years may fail to detect late-emerging effects, such as progressive ovarian reserve decline or delayed spermatogenic recovery. Another important limitation was the absence of standardized antral follicle count assessment. Consequently, the observed reductions in ovarian volume could not be directly correlated with the number of remaining antral follicles or used as definitive evidence of primordial follicle depletion. Reliance on surrogate markers like FSH and AMH may overlook subclinical impairments. Statistical power was limited for subgroup analyses, such as radiation dose effects. In addition, pubertal status at diagnosis was not systematically recorded due to the retrospective design, precluding adjustment for baseline pubertal maturation; as some patients may have already completed puberty before treatment, findings related to pubertal growth and development (e.g., final height, age at menarche) should be interpreted with this limitation in mind. These limitations indicate the need for larger studies to validate and expand upon our observations, as echoed in recent reviews like Anderson et al. (45), which emphasize AMH's role in monitoring ovarian reserve post-treatment and advocate for longitudinal assessments to capture dynamic recovery patterns.

## Conclusion

This study demonstrates a high burden of gonadal dysfunction in childhood sarcoma survivors, with prevalent abnormalities in ovarian reserve and spermatogenesis, and elevated FSH as a key indicator, particularly in males. Both male and female childhood cancer survivors are at risk for gonadal insufficiency, necessitating comprehensive patient education on issues such as gonadal toxicity, infertility, and reduced sexuality, which can profoundly impact quality of life. High-risk patients should receive detailed information on fertility preservation options prior to treatment, ultimately improving quality of life in this growing survivor population.

## Data Availability

The raw data supporting the conclusions of this article will be made available by the authors, without undue reservation.
